# Exploring the Molecular Mechanisms Underlying the Protective Effects of Microbial SCFAs on Intestinal Tolerance and Food Allergy

**DOI:** 10.3389/fimmu.2020.01225

**Published:** 2020-06-16

**Authors:** Maik Luu, Heide Monning, Alexander Visekruna

**Affiliations:** Biomedical Research Center, Institute for Medical Microbiology and Hygiene, Philipps-University Marburg, Marburg, Germany

**Keywords:** commensal bacteria, microbial metabolites, short-chain fatty acids, gut homeostasis, food allergy

## Abstract

A body of evidence suggests that food allergy (FA) has increased in prevalence over the past few decades. Novel findings support the hypothesis that some commensal bacteria and particularly microbial metabolites might contribute to development of oral tolerance and prevention from FA. Recently, beneficial effects of short-chain fatty acids (SCFAs), the main class of gut microbiota-derived metabolites, on FA have been proposed. The intestinal SCFAs are major end products during bacterial fermentation of complex and non-digestible carbohydrates such as dietary fiber. The multifaceted mechanisms underlying beneficial effects of SCFAs on the mucosal immune system comprise the regulation of diverse cellular pathways in epithelial, dendritic, and T cells, as well as the impact on the immunometabolism and epigenetic status of regulatory lymphocytes. Of note, SCFAs are effective inhibitors of histone deacetylases (HDACs). As a consequence, SCFAs appear to be implicated in attenuation of intestinal inflammation and autoimmune diseases. In this review, we will discuss the recent development in this research area by highlighting the role of the individual SCFAs acetate, propionate, butyrate, and pentanoate in promoting the differentiation of regulatory T and B cells and their potential beneficial effects on the prevention of FA. In this context, targeted alterations in the gut microbiota in favor of SCFA producers or supplementation of medicinal food enriched in SCFAs could be a novel therapeutic concept for FA.

## Introduction

The human gut harbors one of the densest microbial habitats on the planet Earth containing thousands of uncharacterized metabolites. Intestinal microbiota synthesize diverse small molecules that play an important role in the communication between the host immune system and commensals (1, 2). Such soluble messengers may affect various physiological processes such as inhibition of colonization of pathogenic bacteria, supporting metabolic and immunological functions of the host, and even the modulation of host behavioral processes (3, 4). Bacterial fermentation of dietary fiber results in the generation of the main class of gut-microbiota derived metabolites, short chain fatty acids (SCFAs). SCFAs, including acetate, propionate, butyrate, and pentanoate, regulate multiple aspects of human health including beneficial effects on autoimmune and inflammatory disorders (5, 6). While host digestive enzymes in the oral cavity, stomach, and upper intestine lack the ability to digest complex carbohydrates such as pectin and inulin, those water-soluble dietary fiber are readily fermented in the gut lumen by various members of the human microbiota. Amounts of SCFAs vary along the gastrointestinal tract reaching the highest concentrations within millimolar range in the proximal colon and cecum (7). Specific bacterial species implicated in the synthesis of individual SCFAs have been recently identified (2). The most dominant commensal butyrate producers belong to the phylum Firmicutes, whereof Clostridia from the human gut microbiota are the major butyrate-producing class (8). Particularly, *Faecalibacterium prausnitzii, Eubacterium rectale*, and several *Roseburia* species are able to synthesize high amounts of this SCFA (9, 10). In contrast to conventionally raised mice that have high levels of acetate, propionate and butyrate, germ-free animals are completely devoid of SCFAs. There is substantial evidence that SCFAs have various effects on host physiology not only in the gut, but also in the distal organs such as brain and lung (11–13). This review summarizes recent work carried out over the past several years illustrating diverse impacts of SCFAs and dietary fiber on host immune system, microbial, and oral tolerance, as well as their beneficial effects on food allergy.

## Mechanisms of SCFA-Mediated Regulation of the Host Immune System

Proposed mechanisms underlying SCFAs-mediated modulation of the gut epithelium and mucosal immune system comprise at least three different modes of action. SCFAs act as diffusible signaling molecules that have substantial effects on eukaryotic cells expressing G protein-coupled receptors (GPRs) such as GPR41, GPR43, and GPR109a (14). Although the preferential binding of individual SCFAs to various GPRs has not yet been completely elucidated, diverse signaling cascades can be activated following ligation of microbial SCFAs to metabolite-sensing molecules. In colonic epithelial cells, propionate and acetate have been shown to induce p38 and ERK MAPK activation through GPR41 and GPR43 (15). These cell surface SCFA-receptors are expressed not only on the gut epithelium, but also on intestinal immune cells such as dendritic cells (DCs) and regulatory T cells (Tregs). The GPR109a expression on DCs supports the proliferation of Tregs and thus promotes tolerogenic effects in the gut (16). In addition, colonic Tregs express high levels of the SCFA-sensing receptor GPR43, which enables them to protect mice against colitis (17). Moreover, SCFA-derived atoms serve as carbon source for epithelial cells, thus directly fueling host metabolism (2). Finally, as strong histone deacetylase (HDAC) and lysine deacetylase (KDAC) inhibitors, butyrate and propionate elicit most of their effects by modulating the expression of various genes involved in several biological processes such as cell proliferation and differentiation, antimicrobial immunity, integrity of epithelial barrier, and intestinal tolerance to bacterial antigens and dietary proteins (18–20). Although some controversies remain, recent findings have revealed that SCFAs enhance the glycolytic rate of immune cells and increase acetyl-CoA concentrations, thus connecting the cellular metabolism and chromatin modifications (13, 21). The SCFA-mediated increase in glucose-derived pyruvate and acetyl-CoA levels in eukaryotic cells leads to the accumulation of citrate, its transport to the cytosol and subsequent conversion into cytosolic acetyl-CoA by ATP citrate lyase (ACLY). ACLY is the key cytosolic enzyme that converts citrate to acetyl-CoA, which is needed for histone acetyltransferase (HAT)-dependent histone acetylation (22). There is a substantial body of evidence that SCFAs are not only HDAC inhibitors, but they are also able to promote histone modifications in immune cells by acting as acyl-CoA precursors. Thus, the carbon atoms derived from SCFAs can directly be transferred to histones via a metabolic-epigenetic link leading to HAT-mediated histone acetylation and recently described histone propionylation and butyrylation (23). Remarkably, SCFAs seem to be unique molecules able to regulate the gene expression at the epigenetic levels by modulating the activity of both, HATs and HDACs. Although further studies are still required to better understand interactions between microbial metabolites, HAT activity and histone acylations, current data suggest that SCFAs provide a pool of acyl groups for generation of acetyl-CoA and other endogenous metabolites in gut epithelial and immune cells, which can be used for various cellular activities (24).

## SCFAs Actively Support the Tolerance to Food Antigens and Commensal Bacteria

Metabolomic analysis of the gut microbial community has shown that SCFAs, a major group of bacterial molecules in the gut lumen, are potent modulators of the mucosal immune system (2, 25). Recent studies have demonstrated that SCFAs are not only locally protective in the intestinal environment, but they can even act in remote tissues such as pancreas, lung, and brain (11, 26, 27). Although it is well-appreciated that SCFAs impact on the colonic epithelial cells, Tregs and DCs, less is known about the complex mechanisms underlying bidirectional interactions between intestinal cellular networks and individual members of SCFA-producing microbiota. Moreover, despite some promising results obtained in experimental murine models, a possible protective effect of SCFAs and dietary fiber on the onset of human gastrointestinal disorders such as inflammatory bowel disease (IBD), celiac disease, and food allergy is relatively poorly characterized. Among SCFAs, butyrate has been specifically associated with the expansion of mucosal Tregs and it also acts as a preferred carbon source for colonocytes (2). During gut homeostasis, the metabolism of colonic epithelial cells is profoundly dependent on oxidative phosphorylation, which leads to high oxygen consumption. Interestingly, microbiota-derived butyrate utilized by the gut epithelium affects the O_2_ levels in these cells resulting in activation of the oxygen sensor hypoxia-inducible factor (HIF), a transcription factor that is crucial for coordinating gut integrity and barrier protection (28). In addition, butyrate and other SCFAs have a strong influence on tight junctions (TJ) and production of mucin (29, 30). Furthermore, SCFAs seem to maintain intestinal barrier function by stimulating the synthesis of antimicrobial peptides and the cytokine IL-18, which strengthens the tolerance to commensal bacteria and promotes intestinal homeostasis (31, 32). Butyrate influences intestinal CD103^+^ DCs by stimulating the GPR109a cell surface receptor, which enables this tolerogenic DC subpopulation to trigger proliferation and expansion of regulatory T cells (Tregs) in mesenteric lymph nodes (16). DCs treated with butyrate, propionate, and pentanoate exhibit a lower capacity to stimulate effector CD4^+^ T cells (33). Small intestinal DCs display a selective capability to induce retinoic acid (RA)-dependent increase in the activity of aldehyde dehydrogenase (ALDH) that strongly supports the tolerance to food antigens due to concomitant expansion of food antigen-reactive Tregs (34). Recently, Surh and colleagues were the first to show that small intestinal Tregs recognize dietary antigens and limit undesired and adverse reaction to food by promoting dominant immunosuppressive response (35). Dietary antigens derived from solid food share the space inside the small intestinal lumen with various dietary components and microbial metabolites. Collectively, diet-derived RA and microbiota-derived SCFAs seem to act synergistically on intestinal DCs to control immune response to food antigens by dampening induction of inflammatory cytokines as well as by inducing Tregs that play a pivotal role in controlling the tolerance to food and commensal antigens (36).

Recent findings have revealed a broad heterogeneity of mucosal Tregs (37, 38), however, there is still no evidence that SCFAs and SCFA-producing bacteria might preferentially support the generation of a particular Treg subpopulation under certain environmental conditions. Butyrate has been suggested to potentiate the expansion of intestinal Tregs by promoting the acetylation of histones at the Foxp3 gene, but also by protecting the Foxp3 protein from degradation through enhancing its acetylation (39). Thus, by acting within Tregs as a KDAC inhibitor to enhance acetylation of Foxp3 protein and as a HDAC inhibitor at the Foxp3 gene locus, butyrate suppresses inflammation and adverse immune responses in intestinal tissues. Beyond modulating the epigenetic status of Tregs, butyrate and other SCFAs have been shown to influence the function of B cells in Peyer′s patches (PPs) and the small intestine. SCFAs appear to be capable of increasing the number of IgA-secreting lamina propria plasma cells and B cells in PPs (21, 40). These effects of SCFAs on B cells seem to be mediated by enhancing their metabolic activity. It has also been suggested that particularly pentanoate and butyrate are able to induce IL-10 production in B cells, which promotes the differentiation of regulatory B cells (Bregs) (13). These unexpected results suggest that microbiota-derived SCFAs are not only important for the maintenance and expansion of mucosal Tregs and their function, but also for promotion of the Breg cell phenotype. Gaining a better understanding of the anatomic sites at which SCFAs-mediated effects on T and B lymphocytes occur under physiological conditions could be of importance for the future. There is some evidence that not only surface molecules of commensal bacteria, but also soluble microbial metabolites such as SCFAs support the synthesis of protective IgA and IgG antibodies during the intestinal infection with *Citrobacter rodentium* by increasing activity of mTOR and glycolysis in B cells (21). This suggests that SCFAs do not only promote the tolerance to food antigens and microbiota by modulating IgA antibody responses, but they also may help eliminating intestinal pathogenic infections.

## Effects of SCFAs and Dietary Fiber on Mast Cells and Food Allergy

**Figure 1:**
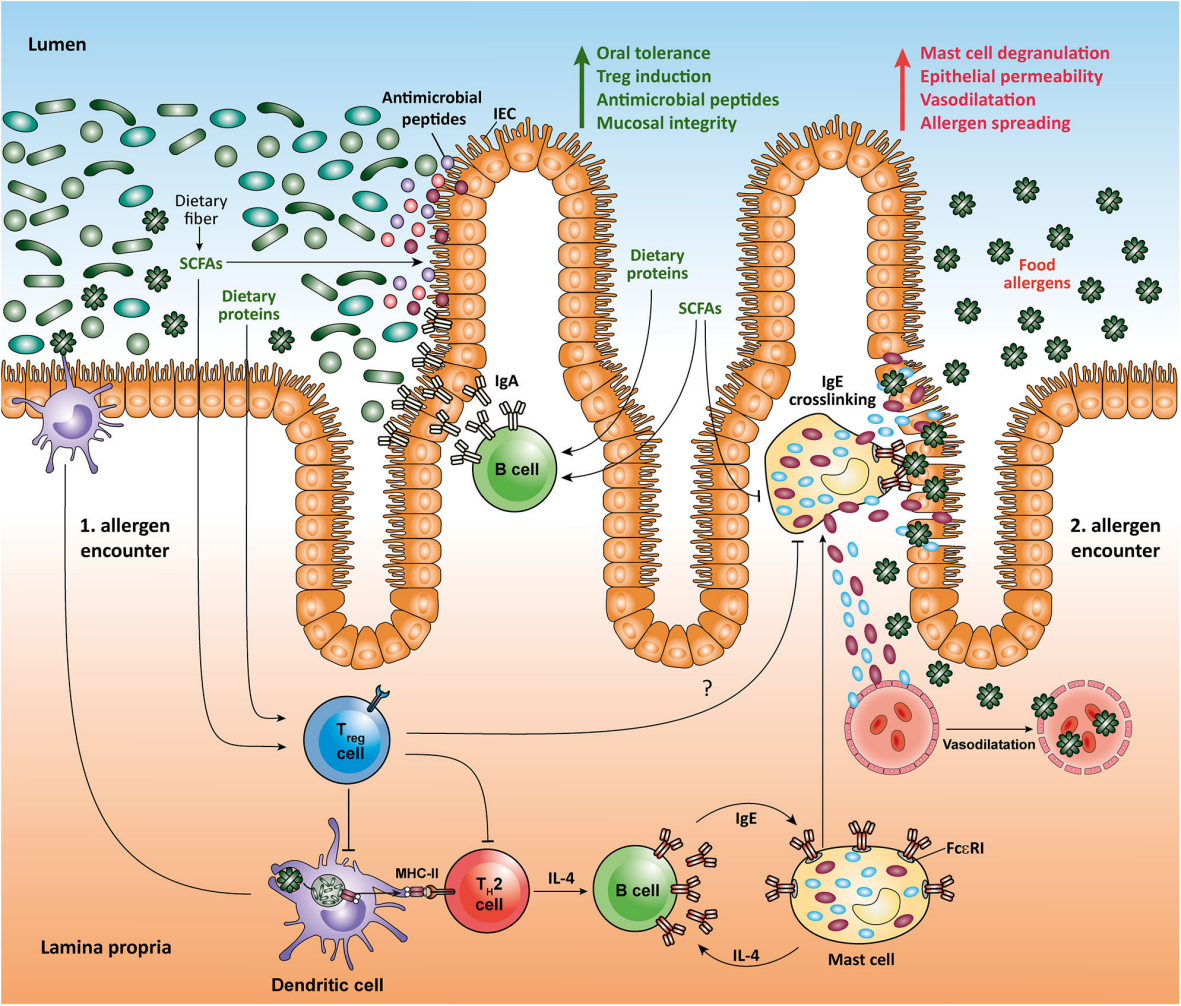
Impact of microbial SCFAs on intestinal homeostasis and food allergy.

A better understanding of the influence of gut-microbiota derived molecules on the maturation and function of the immune system in the small intestine may open novel important therapeutic options in a variety of gastrointestinal disorders ranging from IBD to food allergy. In the last decades, a significant increase in the prevalence of food allergies that is characterized by adverse immune responses to food antigens, which are mainly derived from peanuts, milk, eggs, tree nuts, strawberries, or shellfish, has been observed (41). The most characteristic form of food allergy is mediated by IgE-dependent pathways (42). Human IgE-triggered peanut allergy is associated with a high cell number of somatically mutated and clonally expanded gastrointestinal allergen-specific IgE^+^ B cells suggesting a local isotype switching, which likely includes the transition between IgA and IgE antibody isotypes (43). Recent data suggest that some dietary components such as RA (an active derivative of dietary vitamin A) and dietary peptides, as well as microbial SCFAs may act together to promote intestinal homeostasis and suppress food allergy. Interestingly, dietary proteins induce the expansion of food protein-reactive Tregs in the small intestine, as well as the production of IgA and generation of follicular helper T (Tfh) cells in the PPs, thus strengthening intestinal homeostasis (35, 44, 45). In a mouse model of peanut allergy, SCFAs and RA have been shown to shape local immune responses and oral tolerance by increasing the function of tolerogenic CD103^+^ DCs that are essential for generation of mucosal Tregs. Moreover, high-fiber diet and SCFA supplementation protected mice from food allergy by promoting production of IgA in small intestinal lamina propria and by enhancing the frequency of follicular T (Tfh) cells in the PPs. Particularly, mice orally treated with the SCFAs acetate and butyrate displayed a reduction in anaphylactic clinical scores and diminished serum IgE levels as compared to control animals following induction of peanut allergy (34). In human food allergy, individuals exposed to food allergens have a high amount of intestinal Th2 cells as well as type 2 innate lymphoid cells (ILC2) that produce cytokines such as IL-4, IL-5, and IL-13 (46). IL-4 is known to strongly support the differentiation of B cells into IgE-synthesizing plasma cells (47). The subsequent exposure of those individuals to food allergens mediates the cross-linking of allergen-specific IgE via FcεRI on mast cells. This induces degranulation and release of histamine and several other effector mediators, which results in immediate allergic reaction (41). Interestingly, recent studies suggest that the SCFA butyrate exhibits a direct effect on mast cells by epigenetically regulating the FcεRI-mediated signaling molecules (48–50). Thus, by directly inhibiting the IgE-mediated mast cell degranulation and allergen-induced histamine release, microbial SCFAs such as butyrate could have therapeutic benefits in human food allergies (Figure 1). Of note, high levels of SCFAs butyrate and propionate in feces in early life of children are associated with protection against food allergy and asthma (51). Furthermore, children with cow's milk allergy were shown to have reduced fecal levels of butyrate compared to healthy controls (52). Together, novel results establish an important role for dietary fiber and SCFAs in promoting the integrity of epithelial barrier, oral tolerance and protection against food allergies. These observation could, at least in part, be explained by inhibitory effects of SCFAs on HDACs in several immune cells such as Tregs, B cells, and mast cells, as well as via stimulation of SCFA-receptors such as GPR41, GPR43, and GPR109a on epithelial cells and CD103^+^ DCs.

## Conclusions

Although some controversies remain, accumulating evidence supports the role of microbiota-derived metabolites in promoting tolerogenic immune responses in the healthy intestine. In the last decade, a better understanding of microbiota-interactions that influence many aspects of human health including protection against pathogens, strengthening epithelial barrier function and promotion of tolerance to food antigens and commensals has led to the idea that a healthy core microbiome and its main metabolites SCFAs may be of high therapeutic interest. Such low-cost and potent small molecules might not only help maintaining intestinal homeostasis in healthy individuals, but they could be also applied to a variety of gastrointestinal disorders ranging from IBD and celiac disease to pathogenic conditions such as food allergies and irritable bowel syndrome (IBS), which are often associated with altered gut microbiota. We suggest that designing medicinal food enriched in SCFAs may lead to development of novel therapeutic approaches in food allergy.

## Author Contributions

All authors listed have made a substantial, direct and intellectual contribution to the work, and approved it for publication.

## Conflict of Interest

The authors declare that the research was conducted in the absence of any commercial or financial relationships that could be construed as a potential conflict of interest.
